# Safety and efficacy of tirofiban after intravenous thrombolysis with urokinase in patients with acute ischemic stroke

**DOI:** 10.3389/fneur.2025.1529331

**Published:** 2025-03-06

**Authors:** Dian Qu, Huanhuan Liu, Zhongming Wei, Yao Cheng, Yutong Fei, Jinghan Xu, Xiuyu Lv, Wendi Li

**Affiliations:** ^1^Department of Neurology, Harbin 242 Hospital, Harbin, China; ^2^Center of Oral Medicine, Harbin 242 Hospital, Harbin, China; ^3^Department of Pharmacy, Harbin 242 Hospital, Harbin, China

**Keywords:** urokinase, acute ischemic stroke, tirofiban, urokinase thrombolysis, efficacy

## Abstract

**Introduction:**

Tirofiban combined with alteplase thrombolysis or endovascular therapy has been proven to improve the prognosis of patients with acute ischemic stroke (AIS). Some patients, due to the extended time window beyond 4 h and economic considerations, opt for urokinase thrombolysis instead of alteplase thrombolysis in China. However, there is currently limited research on the use of urokinase thrombolysis bridged with tirofiban.

**Methods:**

We employed propensity score match to pair 80 sets of patients from a total of 196 individuals who underwent urokinase thrombolysis for acute ischemic stroke. The study analyzed the 14-day National Institutes of Health Stroke Scale (NIHSS), 90-day modified Rankin Scale (mRS), bleeding events, and compared the odds ratio (OR) of patients with mRS scores of 0–2 within the subgroups.

**Results:**

The results show that the NIHSS at 14 days of the tirofiban group was significantly lower than that of the dual antiplatelet group. No significant difference was found in the proportion of patients with mRS score 0–2. The odds ratios were slightly different in subgroups classified with or without previous stroke and hypertension.

**Discussion:**

It was confirmed that the tirofiban might be safe in AIS patients received tirofiban after urokinase thrombolysis and could improve short-term neurological function.

## Introduction

Urokinase has been approved as a promising alternation in the treatment of acute ischemic stroke within 6-h after onset according to the work of Cooperating Group for National “95” Project and the Chinese guideline for acute ischemic stroke 2018 (1). The rate of early neurological deterioration occurring after thrombolytic therapy of patients in Asia is 15.9% (95% CI: 7.4–24.5%) (2). There is ongoing active investigation into the use of different antiplatelet agents following intravenous thrombolysis in order to prevent platelet aggregation and subsequent vascular reocclusion (3). GP IIb/IIIa antagonists have the ability to reduce thrombus growth and to improve flow in cerebral microcirculation. Results of medial cerebral arteria occlusion models with autologous clots showed that complete recanalization rate increased 33% when GP IIb/IIIa antagonists combined with rt-PA compared with antagonists alone (4). Several clinical trials indicated that tirofiban in combination with rt-PA or endovascular intervention was associated with good functional outcomes at 3 months, and not associated with a higher rate of intracranial hemorrhage (5, 6). Most previous studies have focused on the safety and efficacy of bridging to tirofiban after alteplase thrombolysis (7). Only few studies have addressed the use of intravenous tirofiban after thrombolysis with urokinase. The combination of intravenous tirofiban with intra-arterial urokinase and mechanical thrombolysis was proved to success in reperfusion of the occluded artery without increasing the hemorrhagic risk and with good functional outcome (8, 9). Patients receiving alteplase and those receiving urokinase for intravenous thrombolysis had comparable Outcomes, while the latter had a higher risk of extracranial bleeding. Even so, urokinase is a good option for patients who cannot afford tirofiban (10). Liu et al. (11) observed early administration of tirofiban after urokinase thrombolysis improved the long-term prognosis for patients with branch atheromatous disease. Given the widespread use of urokinase in developing countries and its 6 h thrombolytic time window, we try to characterize the outcomes of bridging with tirofiban in patients using urokinase thrombolysis within 4–6 h of onset through retrospective analysis.

## Methods

### Patient selection

**Figure 1:**
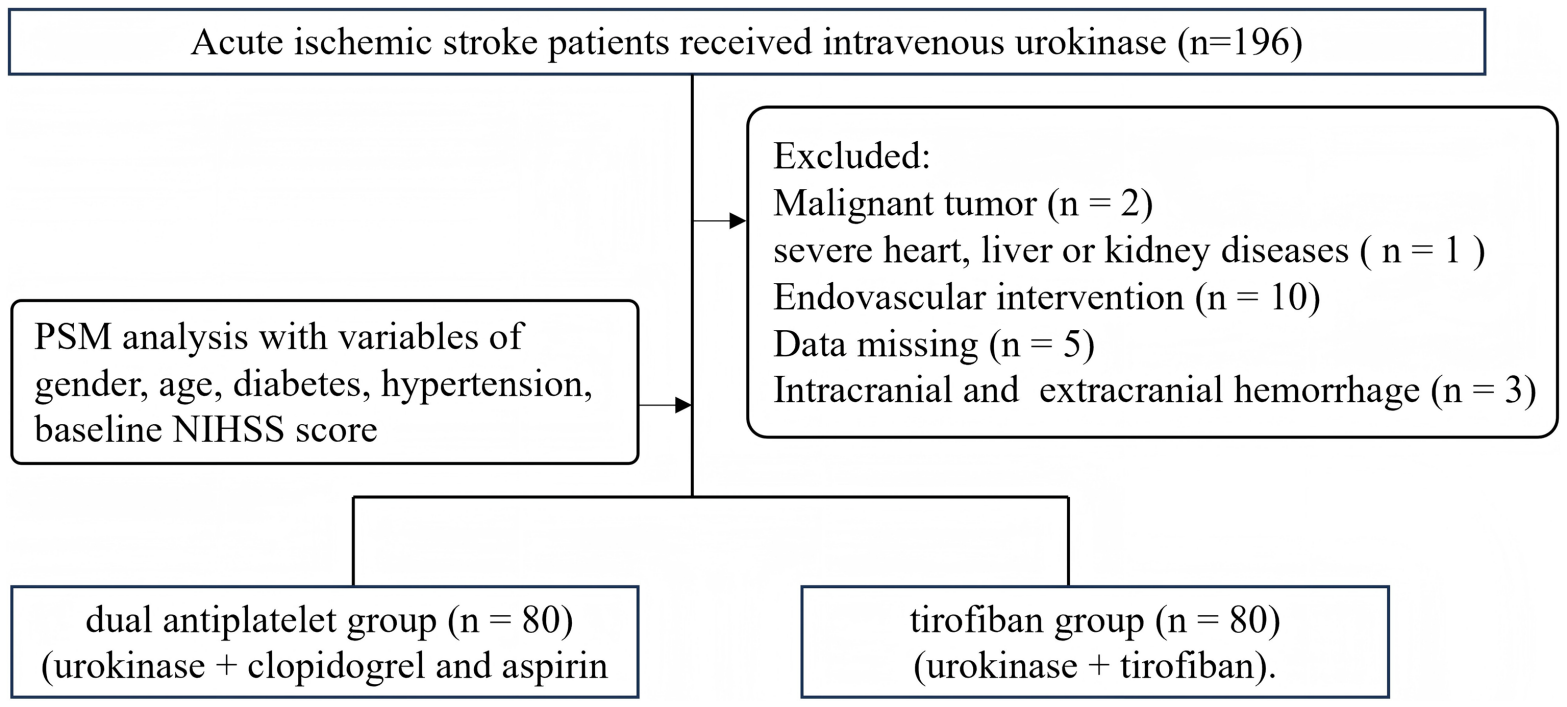
Flow chart of patient screening.

The study retrospectively collected a total of patients (*n* = 196) with AIS who received intravenous urokinase within 4–6 h of onset between March 2019 and June 2023 in the Department of Neurology of Harbin 242 Hospital. All their urokinase thrombolysis followed the Chinese Guidelines for Diagnosis and Treatment of Acute Ischemic Stroke 2018 (12). We excluded patients who did not have a complete medical history, those who had critical basic diseases (malignant tumor, severe heart, liver or kidney diseases), those who required interventional therapy after thrombolysis and those who were lost during follow-up. All patients underwent brain CT after thrombolysis to ensure no bleeding in the brain or bleeding elsewhere (Figure 1). The study was approved by the Institutional Ethics Committee of Harbin 242 Hospital and written informed consent was obtained from all participants.

### Treatments

For onset of ischemic stroke within 4–6 h, intravenous thrombolysis with urokinase (20,000 units/kg, maximum dose of 1.5 million IU) was considered, which was dissolved in 100 mL of normal saline and maintained through intravenous drip for half an hour. According to whether they received tirofiban early after thrombolytic therapy, patients were divided into dual antiplatelet group (urokinase + clopidogrel and aspirin) and tirofiban group (urokinase + tirofiban). Patients in the dual antiplatelet group were given clopidogrel 75 mg/day and aspirin 100 mg/day 24 h after urokinase thrombolysis, followed by clopidogrel and aspirin for a total of 3 months. For patients in the tirofiban group, the initial 30 min intravenous infusion rate of tirofiban was 0.4 μg/kg/min, within 2–24 h after urokinase thrombolytic therapy, followed by 0.1 μg/kg/min intravenous infusion for 24 h–72 h. After the first dose of aspirin 100 mg and clopidogrel 75 mg were given 4 h before tirofiban was discontinued, administration was continued for 3 months.

### Outcome measures

Basic demographic information was collected from the healthcare system. Blood pressure, blood glucose and lipids, platelet, coagulation indicators, NIHSS and modified Rankin Scale (mRS) scores were evaluated after admission. Clinical outcomes were assessed by a stroke neurologist using NIHSS score at 14 days and mRS at 90 days. A long-term neurological improvement was defined as a modified Rankin Scale score of 0 to 2. Intracranial hemorrhage (ICH), symptomatic ICH, extracranial bleeding and all-cause mortality were studied as safety outcomes.

### Statistical analysis

The IBM SPSS version 22.0 was used for all statistical analyses and two-sided *p*-values <0.05 was considered significant. Patients in the two groups were matched 1:1 based on propensity score match (PSM) using the nearest neighbor matching algorithm with a caliper width of 0.02. Patient characteristics were presented as mean ± standard, median (interquartile range) or count (percentage) as appropriate. Independent sample *t*-test, the Mann–Whitney *U* test, *χ*^2^ test or Fisher exact test were used according to the data types. Odds ratios (ORs) and corresponding 95% confidence intervals (CIs) were estimated accordingly with the dual antiplatelet group being the reference group. Subgroup treatment effects were tentatively analyzed.

### Results

This study enrolled 196 patients (age: 59.56 ± 10.64 years; male: 135, 68.9%) who received intravenous urokinase for AIS. Eighty matched pairs were found by the PSM analysis, the covariates of which included gender, age, diabetes, hypertension, baseline NIHSS score. Eighty patients were enrolled in both the tirofiban group and the dual antiplatelet group (age: 60.58 ± 10.3 years vs. 60.0 ± 9.0 years; male %: 68.8% vs. 70.0%). The incidence of hypertension was similar in both groups (65% vs. 63.7%, *p* = 0.869), as was the incidence of diabetes (38.8% vs. 42.5%, *p* = 0.629). No significant differences were found between the basic clinical characteristics of the patients from the two groups (Table 1).

**Table 1:** Comparison of basic data between tirofiban group and dual antiplatelet group after PSM.

	Tirofiban group (*n* = 80)	Dual antiplatelet group (*n* = 80)	*p*-value
Age, year	60.58 ± 10.3	60.0 ± 9.0	0.702
Male, *n* (%)	55 (68.8%)	56 (70.0%)	0.864
Hypertension, *n* (%)	52 (65%)	51 (63.7%)	0.869
Diabetes mellitus, *n* (%)	31 (38.8%)	34 (42.5%)	0.629
Hyperlipidemia, *n* (%)	11 (13.8%)	17 (21.3%)	0.212
Atrial fibrillation, *n* (%)	7 (8.8%)	3 (3.8%)	0.191
Smoking, *n* (%)	39 (48.8%)	43 (53.8)	0.527
Previous stroke, *n* (%)	14 (17.5%)	13 (16.3%)	0.833
Platelets,10^9^/L	206 (64)	217 (54)	0.357
Low density lipoprotein, mmol/L	2.54 (0.95)	2.35 (1.20)	0.276
HbA1c, %	5.8 (1.50)	6.1 (2.3)	0.092
Onset to admission time, h	5 (1.1)	5 (0.8)	0.796
Baseline mRS score	3 (1)	4 (1)	0.885
Baseline NIHSS score	4 (4)	5 (3)	0.174

Values are mean ± standard deviation (SD) or median (interquartile range). PSM, propensity score match; HbA1c, hemoglobin A1c; mRS, modified Rankin Scale; NIHSS, National Institute of Health Stroke Scale.

**Figure 2:**
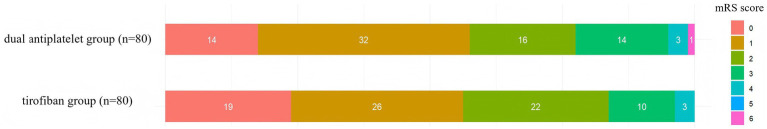
Distribution of mRS scores at 3 months.

The NIHSS at 14 days of the tirofiban group was significantly lower than that of the dual antiplatelet group [1 (3) vs. 3 (2), *p* < 0.001]. However, there was no significant difference in mRS scores at 3 months [tirofiban group vs. dual antiplatelet: 1 (1) vs. 1 (1), *p* = 0.625] and the proportion of patients with mRS score 0–2 [67 (83.8%) vs. 62 (78.5%), *p* = 0.424]. The distributions of mRS scores at 3 months was showed in Figure 2. Five patients in the dual antiplatelet group and one in the tirofiban group had worsening NIHSS at 14 days that were higher than that at admission (Fisher: *p* = 0.210). Neither group had any cases of intracranial hemorrhage within 7 days. In the tirofiban group, there were three cases of extracranial hemorrhage (two patients had gingival bleeding and one patient had hematuria), while the dual antiplatelet group had one patient with gingival bleeding (Table 2). Among the 196 patients treated with urokinase thrombolysis, 10 (5.1%) experienced hemorrhagic events. Of these, three cases (including one intracranial hemorrhage) were subsequently excluded from antiplatelet therapy, while an additional three cases (two gastrointestinal bleeding and one gingival bleeding) were not included in the propensity score matched study groups. During the follow-up period of 6 months, three patients from the tirofiban group and one patient from the dual antiplatelet group died.

**Table 2:** Outcomes and treatment complications in patients treated with tirofiban or dual antiplatelet.

	Tirofiban group (*n* = 80)	Dual antiplatelet group (*n* = 80)	*p*-value
NIHSS at day 14	1 (3)	3 (2)	**<0.001**
mRS at 3 months	1 (1)	1 (1)	0.625
ICH within 7 days, *n* (%)	0 (0%)	0 (0%)	—
Extracranial bleeding within 7 days, *n* (%)	3 (3.8%)	1 (1.3%)	0.620
Death from any cause within 6 months, *n* (%)	3 (3.8%)	1 (1.3%)	0.620
Recurrent cerebral infarction within 6 months, *n* (%)	0 (0%)	2 (2.5%)	0.487

Values are median (interquartile range). mRS, modified Rankin Scale; NIHSS, National Institute of Health Stroke Scale; ICH, intracranial hemorrhage. The bold value represents *P* < 0.05, which is statistically significant.

**Figure 3:**
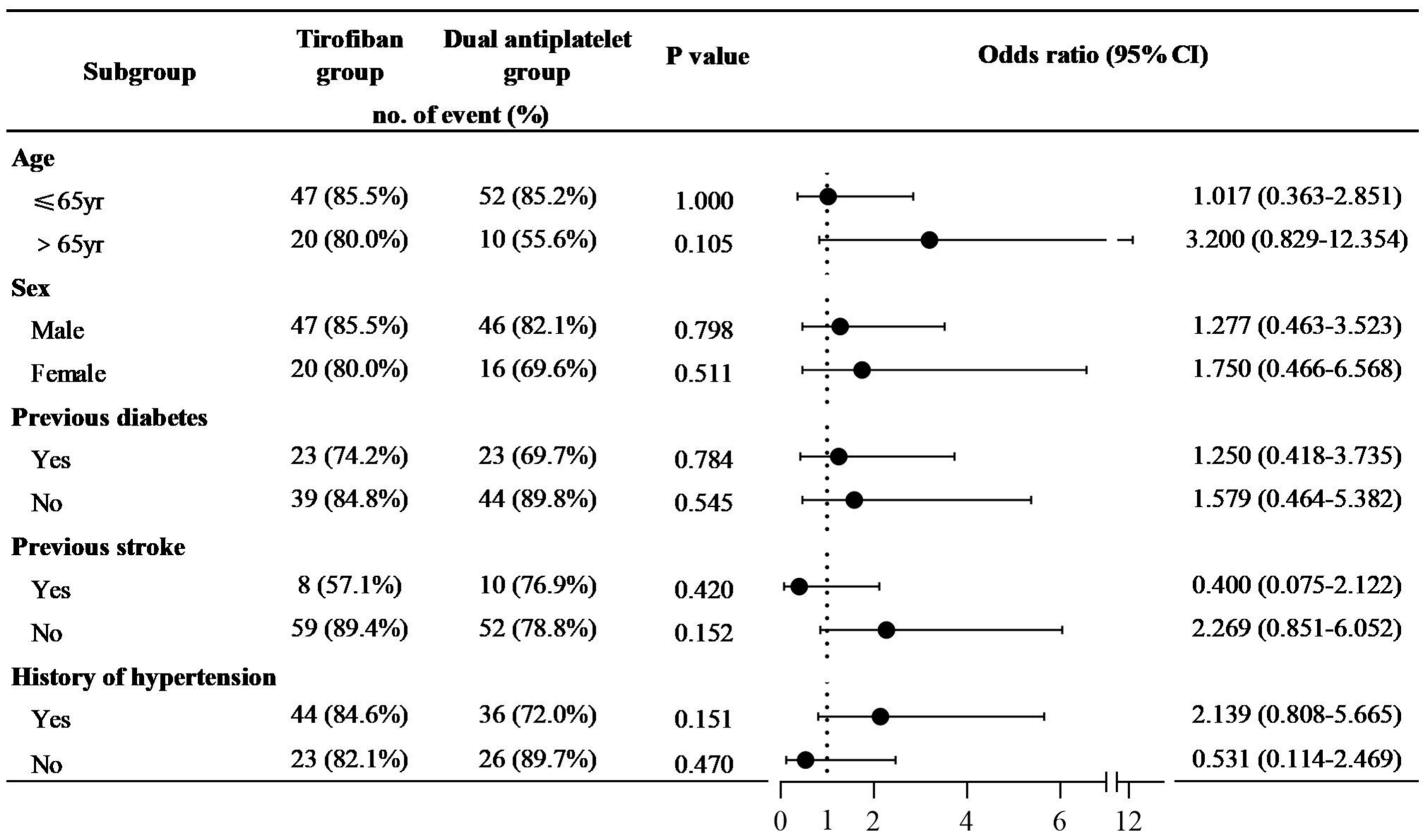
Effect of treatment with tirofiban or dual antiplatelet on proportion of 0–2 mRS in different baseline characteristics patients. The ratio of long-term neurological improvement (mRS 0–2) among different treatment regimens is compared in different subgroups. *p*-values, odds ratios (OR), and their 95% confidence intervals are showed in figure. The dashed line represents the reference line where the OR equals 1, while the points and lines indicate the OR values and their corresponding confidence intervals, respectively. There was no statistically significant difference among the subgroups.

Moreover, functional independence (mRS 0–2) was compared between patients treated with tirofiban or dual antiplatelet by subgroup analysis of different baseline characteristics (Figure 3). No statistically significant differences were observed across the subgroups. In stratified analyses by age, stroke history, and hypertension status, tirofiban treatment demonstrated a non-significant trend toward potentially favorable long-term outcomes in specific subgroups. For patients older than 65 years compared to those 65 years or younger, the odds ratios were OR = 1.017 (95% CI, 0.363–2.851) vs. OR = 3.200 (95% CI, 0.829–12.354). When analyzing by history of stroke, the odds ratios were OR = 0.420 (95% CI, 0.075–2.122) for patients with a history of stroke compared to OR = 2.269 (95% CI, 0.851–6.052) for those without, and for hypertension, the odds ratios were OR = 2.139 (95% CI, 0.808–5.665) for patients with hypertension versus OR = 0.531 (95% CI, 0.114–2.469) for those without.

## Discussion

Our study found that intravenous tirofiban treatment within 24 h after urokinase thrombolysis did not increase the risk of bleeding, and the NIHSS score was lower at 14 days in patients with AIS at 4–6 h after onset, while no significant difference was found in mRS score at 3 months. Long-term neurological improvement ratios of patients were inconsistent in the subgroups of patients with stroke or hypertension.

Previous studies on the safety of tirofiban are similar to those in this paper. A meta-analysis including 17 studies with 2,914 AIS patients showed tirofiban via intra-arterial administration was associated with increased risk of fatal ICH (OR, 2.90; 95% CI, 1.12–7.55; *p* = 0.03), while intravenous administration was not (13). In patients with thrombolysis of alteplase plus tirofiban, Zhou et al. (14) and Zhang et al. (15) also found tirofiban did not increase the risk of any ICH and mortality. Moreover, tirofiban administered at 2–12 h after alteplase thrombolysis were beneficial and safe (16). A small randomized controlled study found that thrombolysis of urokinase plus tirofiban could improves self-care ability in AIS patients without clear criminal vessels by relieving the level of inflammatory factors (17). Considering the pharmacokinetics of tirofiban, which has a short half-life of 2 h and platelet aggregation was restored to approximately 50% by 4 h after drug withdrawal, tirofiban is safe for AIS patients after intravenous thrombolysis. Our study verified its safety in AIS patients with urokinase thrombolysis within 4–6 h of onset and explored the application scenarios of tirofiban.

The safety of urokinase plus tirofiban was proved; however, long-term functional improvements in this study do not differ between the two groups. The Safety of Tirofiban in acute Ischemic Stroke trial also corroborated the safety of tirofiban in hemorrhagic complications without significant differences in functional improvement (18). Tao et al. (19) reached the same conclusion when comparing tirofiban and dual antiplatelet in non-thrombolytic AIS patients by prospective non-randomized study. In patients with AIS without large or medium-sized vessel occlusion, the percentage of patients with a score of 0 or 1 on the mRS at 90 days was 29.1% with tirofiban and 22.2% with aspirin (*p* = 0.02). The article identifies patients suitable for receiving tirofiban: those with acute cerebral infarction who are not candidates for thrombolysis and without large or medium-sized vessel occlusion and patients experiencing symptom progression after thrombolysis (20). In patients who were not undergoing alteplase thrombolysis or endovascular thrombectomy therapy at the early stage, the proportion of favorable functional outcomes was higher in the tirofiban group (79.1%) than that in the control group (67.8%) at 90 days (*p* = 0.0155) (21). In our study, the inconsistency between the NHISS at day 14 and mRS at 3 months in this study may be due to the participants most of which had mild strokes and our control group patients took dual antiplatelet therapy rather than aspirin monotherapy. From the perspective of assessment criteria, the NIHSS score focuses on acute neurological deficits and demonstrates high objectivity. Conversely, the mRS score evaluates long-term functional independence and tends to be more subjective. Evaluating long-term prognosis can be influenced by various factors, including complications, rehabilitation, and psychosocial aspects. Unfortunately, we did not collect this part of the data during the follow-up. We hypothesize that tirofiban may accelerate recovery during the acute phase without altering the ultimate recovery ceiling.

Tirofiban inhibits the final common pathway to platelet aggregation by reversibly blocking fibrin binding receptors, aspirin and clopidogrel rely on either inhibiting prostaglandin synthesis or blocking signal transduction pathways (7, 22). If the NIHSS scores of 24 h, 48 h and 72 h after thrombolysis had been collected previously, it would be helpful to identify the short-term effect of tirofiban and whether it can reduce early reocclusion rate. Given the retrospective nature of this analysis and missing data of above in the medical records, our results might underestimate the expected effect of tirofiban. So far, the recommended dosage of tirofiban is 30-min loading infusion at a rate of 0.4 μg/kg/min followed by a continuous infusion of 0.1 μg/kg/min (23). No uniform time standard for the use of tirofiban after thrombolysis at has been established. We conducted subgroup analyses for the duration of tirofiban treatment (24 h, 48 h, 72 h) and found it was not associated with prognosis (NIHSS at 14 days: *F* = 0.481, *p* = 0.620). As for the timing of tirofiban initiation, one study explored the efficacy of tirofiban starting at different times after alteplase thrombolysis. Patients were divided into three groups according to the time points of tirofiban administration: Group A (2 h), Group B (2–12 h), Group C (12–24 h). The efficacy in Group A was better than that in Group C (*p* = 0.006) and no significant difference in the efficacy was found between Groups A and B (*p* = 0.268) (13).

The types of stroke suitable for tirofiban treatment are small vascular disease, rather than large vascular occlusion, and progressive stroke (6). The characteristics of applicable patients are the premise of precision medicine. In the subgroups of patients with previous cerebral infarction or without hypertension, the OR values of less than 1 indicate a poor long-term prognosis for patients received tirofiban. However, the 95% CI of the OR values contain 1, which means that the results are not exact. In patients over the age of 65 or those with hypertension, the long-term prognosis is more favorable when treated with tirofiban. This may be attributed to the fact that these patients often exhibit chronic vascular changes, such as endothelial dysfunction and atherosclerosis, which easily leads to platelet aggregation. Tirofiban is more effective in blocking the final pathway of platelet aggregation (24). Therefore, the specific target patients and detailed optimal dosage need to be further validated in prospective, dose-escalating RCTs.

This study has several limitations. As a retrospective case-control study, there may be potential selection bias such as a higher risk of recurrent infarction in tirofiban patients. Additionally, there is a lack of data on NIHSS scores at 48 and 7 days, as well as factors influencing long-term prognosis. In the subgroup analysis, the small sample size may result in non-significant statistical differences, indicating the need for further expansion of case collection.

## Conclusion

Tirofiban might be safe in patients with acute ischemic stroke after intravenous thrombolytic therapy with urokinase. The NIHSS at 14 days of the patients treated with urokinase and tirofiban therapy was reduced obviously, yet long-term functional improvement was not observed. Combination therapy should be used with caution in patients with hypertension and previous cerebral infarction.

## Data Availability

The original contributions presented in the study are included in the article/supplementary material, further inquiries can be directed to the corresponding authors.
